# Spatio-Temporal Distribution of Cell Wall Components in the Placentas, Ovules and Female Gametophytes of *Utricularia* during Pollination

**DOI:** 10.3390/ijms22115622

**Published:** 2021-05-25

**Authors:** Bartosz Jan Płachno, Małgorzata Kapusta, Piotr Świątek, Krzysztof Banaś, Vitor F. O. Miranda, Anna Bogucka-Kocka

**Affiliations:** 1 Department of Plant Cytology and Embryology, Faculty of Biology, Institute of Botany, Jagiellonian University in Kraków, 9 Gronostajowa St., 30-387 Kraków, Poland; 2 Department of Plant Cytology and Embryology, Faculty of Biology, University of Gdańsk, 59 Wita Stwosza St., 80-308 Gdańsk, Poland; malgorzata.kapusta@ug.edu.pl; 3 Faculty of Natural Sciences, Biotechnology and Environmental Protection, Institute of Biology, University of Silesia in Katowice, 9 Bankowa St., 40-007 Katowice, Poland; piotr.swiatek@us.edu.pl; 4 Department of Plant Ecology, Faculty of Biology, University of Gdańsk, 59 Wita Stwosza St., 80-308 Gdańsk, Poland; k.banas@ug.edu.pl; 5 Laboratory of Plant Systematics, School of Agricultural and Veterinarian Sciences, São Paulo State University (Unesp), Jaboticabal CEP 14884-900, SP, Brazil; vitor.miranda@unesp.br; 6 Chair and Department of Biology and Genetics, Medical University of Lublin, 20-093 Lublin, Poland; anna.kocka@umlub.pl

**Keywords:** arabinogalactan proteins, glycoproteins, AGPs, carnivorous plants, embryo sac, integument, Lentibulariaceae, ovule, pollen tube guidance

## Abstract

In most angiosperms, the female gametophyte is hidden in the mother tissues and the pollen tube enters the ovule via a micropylar canal. The mother tissues play an essential role in the pollen tube guidance. However, in *Utricularia*, the female gametophyte surpasses the entire micropylar canal and extends beyond the limit of the integument. The female gametophyte then invades the placenta and a part of the central cell has direct contact with the ovary chamber. To date, information about the role of the placenta and integument in pollen tube guidance in *Utricularia*, which have extra-ovular female gametophytes, has been lacking. The aim of this study was to evaluate the role of the placenta, central cell and integument in pollen tube pollen tube guidance in *Utricularia nelumbifolia* Gardner and *Utricularia humboldtii* R.H. Schomb. by studying the production of arabinogalactan proteins. It was also determined whether the production of the arabinogalactan proteins is dependent on pollination in *Utricularia*. In both of the examined species, arabinogalactan proteins (AGPs) were observed in the placenta (epidermis and nutritive tissue), ovule (integument, chalaza), and female gametophyte of both pollinated and unpollinated flowers, which means that the production of AGPs is independent of pollination; however, the production of some AGPs was lower after fertilization. There were some differences in the production of AGPs between the examined species. The occurrence of AGPs in the placental epidermis and nutritive tissue suggests that they function as an obturator. The production of some AGPs in the ovular tissues (nucellus, integument) was independent of the presence of a mature embryo sac.

## 1. Introduction

In typical angiosperm plants, the female gametophyte (embryo sac) is hidden in the ovule and is covered by the nucellus and one or two integuments [1]. Angiosperm ovules are incredibly diverse in the terms of their size, the degree of ovule curvature, nucellus thickness, number of integuments and their thickness, the formation of the micropyle, the length of the funiculus, the degree of the vascularization of the ovule and the presence of structures such as an endothecium, hypostase and postament [2]. Sporophytic tissues not only protect the female gametophyte and transport nutrients to it, but also guide the male gametophyte to the female gametophyte e.g., [3,4,5,6,7]. The great success of angiosperms is associated with the diverse structure of their styles and ovules and the sites of the pollen tube pathway [8,9,10,11]. During pollen tube growth, there is a dialogue between the male gametophytes and sporophytic tissues [12], but there is also competition between the pollen tubes, i.e., gametophytic competition [9,13]. In most cases, the pollen tube meets the female gametophyte only when it grows into the micropylar canal and the first elements of the female gametophyte that it meets are the synergids. However, there are some exceptions. In *Plumbago* and *Plumbagella* in which synergids are absent, the egg cell takes over the functions of a synergid [14]. Another evolutionary experiment is an extra-ovular female gametophyte, which extends beyond the limit of the integument. These specific partially “naked” female gametophytes have evolved in the different lineages of angiosperms, such as: Santalales: *Thesium*; Gentianales: *Galium*; Cornales: *Philadelphus*; Lamiales: *Torenia*, *Lindernia* and *Utricularia* [15,16,17,18]. Unfortunately, apart from *Torenia* and *Lindernia*, detailed knowledge about the interactions of an extra-ovular female gametophyte with the sporophytic tissues and pollen tubes is quite limited. The model species is *Torenia fournieri* Linden ex E. Fourn. [19,20], which has the extra-ovular egg apparatus and was used to prove that the synergids are responsible for attracting the pollen tubes in angiosperms [21,22,23]. In *Torenia*, the synergids produce small proteins (LUREs), which attract the pollen tubes [24]. However, the ovular arabinogalactan sugar chain (AMOR) is needed to make the pollen tubes fully competent for LURE peptides, and therefore the sporophytic tissues use arabinogalactan proteins to guide the gametophytic pollen tube [20,25].

Arabinogalactan proteins (AGPs) are important signaling molecules during cell–cell communication, which are spatio-temporally distributed in the plant generative organs e.g., [26,27,28] but have been also identified in vegetative organs (in mesophyll cell, epidermal cells, xylem, root cap cells, etc.) [29]. AGPs have been recorded in the styles, ovules and female gametophytes of various plant species. Their occurrence is connected with the specific stages of development, a specific tissue, organ and/or pollen tube growth e.g., [7,28,30,31,32,33]. In some species, the production of AGPs in ovules is triggered by pollen tube passage [6,33,34], while in others, their production is independent of pollination [7].

However, there is still a lack of information about the AGPs in ovules of *Utricularia*. In some species of this genus, the central cell is hypertrophied at the micropylar pole and invades the placenta. Moreover, a part of the central cell has direct contact with the ovary chamber [16,18,35]. According to Khan [36], in *Utricularia*, the pollen tube meets the female gametophyte in the ovarian cavity (exogamous fertilization). Although the occurrences of AGPs in the ovules and female gametophytes have been extensively studied in porogamous species, only one study has addressed this in a plant with a different mode of pollen tube growth, the chalazogamous species *Mangifera indica* L. [37].

Therefore, the aim of this study was to evaluate the role of the placenta (the epidermis and the special “nutritive tissue” that is situated below the embryo sac [38]), the ovular tissues and hypertrophied central cell and the integument in pollen tube guidance in *Utricularia nelumbifolia* Gardner and *Utricularia humboldtii* R.H.Schomb. by studying the production of arabinogalactan proteins. It was also determined whether the arabinogalactan proteins in *Utricularia* are dependent on pollination.

## 2. Results

### 2.1. Arabinogalactan Proteins in the Placenta and Ovules before Pollination

**Figure 1:**
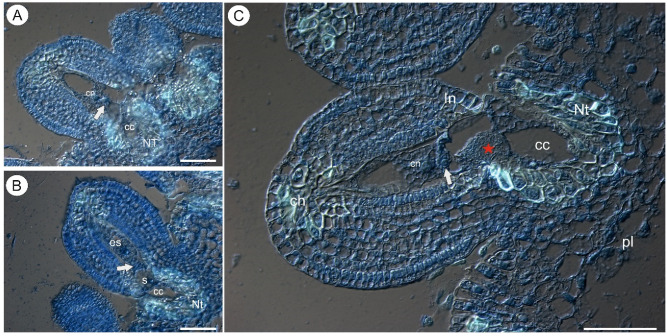
Histology of an ovule of *Utricularia nelumbifolia*; differential interference contrast (DIC) optics. Sections stained with DAPI (blue fluorescence) and DAB (decolorized aniline blue, white fluorescence). (**A**,**B**) Ovules before penetration by the pollen tubes. (**C**) Ovule after penetration by the pollen tube (red star). Note the egg cell (arrow), embryo sac (es), synergid (s), nucleus of the central cell (cn), hypertrophied part of the central cell (cc), placental nutritive tissue (Nt), integument (In), chalaza (ch), placenta (pl). Note callose occurrence (white fluorescence) in the cell walls of the placental nutritive tissue cells and chalaza cells. All bars 50 µm.

**Figure 2:**
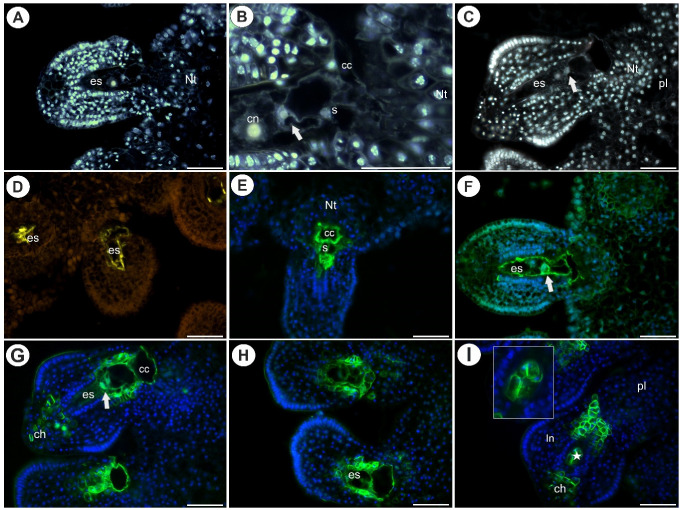
Histology and AGP (JIM13) detection in an ovule of *Utricularia nelumbifolia* and *Utricularia humboldtii* before pollination. (**A**) Section through the ovule and placenta of *U. nelumbifolia* (stained with DAPI, blue fluorescence); note the presence of a mature embryo sac (es), placental nutritive tissue (Nt). (**B**) *U. nelumbifolia* (stained with DAPI, blue fluorescence), higher magnification of the micropylar part of the ovule and embryo sac; note the egg cell (arrow), synergid (s), nucleus of the central cell (cn), hypertrophied part of the central cell (cc), placental nutritive tissue (Nt). (**C**) Section through the ovule and placenta of *U. humboldtii* (stained with DAPI, blue fluorescence); note the presence of a mature embryo sac (es), egg cell (arrow), placental nutritive tissue (Nt), placenta (pl). (**D**–**F**) Arabinogalactan protein (labeled with JIM13, green fluorescence) detected in *U. nelumbifolia*: embryo sac (es), egg cell (arrow), synergid (s), hypertrophied part of the central cell (cc), placental nutritive tissue (Nt); yellowish signal is autofluorescence. (**G**,**H**) Arabinogalactan protein (labeled with JIM13, green fluorescence) detected in *U. humboldtii*: embryo sac (es), egg cell (arrow), hypertrophied part of central cell (cc), placental nutritive tissue (Nt), chalaza (ch). (**I**) Arabinogalactan protein (labeled with JIM13, green fluorescence) detected in the ovule of *U. humboldtii* that did not contain a mature female gametophyte. Note the occurrence of AGPs in remaining nucellus epidermis (star, and framed part), integument (In), chalaza (ch), placenta (pl). All bars 50 µm.

In both species, there were mature embryo sacs in most of the ovules during anthesis (Figure 1A–C and Figure 2A–C). Only some of the ovules did not contain a mature embryo sac (e.g., there was one nucleate embryo sac that had arrested in development). In *Utricularia nelumbifolia* and *U. humboldtii*, there was an intense accumulation of AGPs (labeled with JIM13) in the embryo sac, especially in the micropylar enlarged part of the central cell (Figure 2D–H). Moreover, these AGPs were also detected in the synergids and egg cell (Figure 2E–G). In *U. humboldtii*, there was an intense accumulation of AGPs (labeled with JIM13) in the chalaza cells and integument cells at the micropylar pole (Figure 2G). Only some of the placenta cells had these AGPs. These cells were located near the micropylar part of the embryo sac. There were no AGPs (labeled with JIM13) in the chalazal ovular cells in *Utricularia nelumbifolia*, although they did occur in a few of the integument cells that adhered to the embryo sac (Figure 2D). However, they only occurred in the placental epidermal cells that had contact with central cell (Figure 2E). Nucellus epidermis was persistent even in ovules in which the development of the embryo sac has been stopped (*U. humboldtii*). In those ovules, AGPs (labeled with JIM13) were detected in the nucellus, chalaza and integument (Figure 2I).

**Figure 3:**
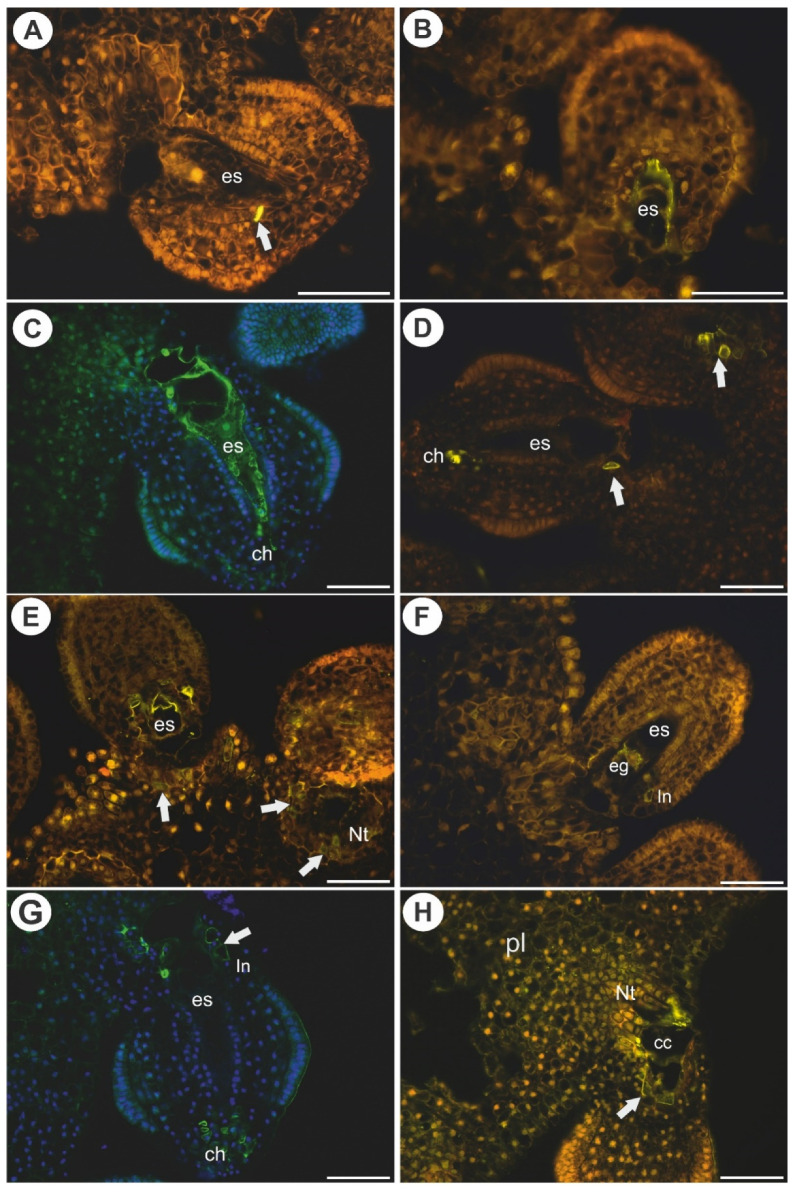
Arabinogalactan proteins (labeled with JIM8 and with JIM14, green fluorescence) detected in *Utricularia nelumbifolia* and *Utricularia humboldtii* before pollination. (**A**,**B**) Arabinogalactan protein (labeled with JIM8, green fluorescence) detected in *U. nelumbifolia*: embryo sac (es), positive signal in the integument cell (arrow); yellowish signal is autofluorescence. (**C**,**D**) Arabinogalactan protein (labeled with JIM8, green fluorescence) detected in *U. humboldtii*, embryo sac (es), chalaza (ch), note the positive signal in the integument cells (arrow); yellowish signal is autofluorescence. (**E**,**F**) Arabinogalactan protein (labeled with JIM14, green fluorescence) detected in *U. nelumbifolia*: embryo sac (es), positive signal in placenta nutritive tissue (Nt) cell (arrow), egg cell (eg), integument (In); yellowish signal is autofluorescence. (**G**,**H**) Arabinogalactan protein (labeled with JIM14, green fluorescence) detected in *U. humboldtii;* embryo sac (es), placenta (pl), nutritive tissue (Nt), integument (In), chalaza (ch), positive signal in the integument cells (arrow); yellowish signal is autofluorescence. All bars 50 µm.

In *Utricularia nelumbifolia*, the JIM8-labeled epitopes were only detected in a single or a few cells of the endothelium. None of these AGPs were detected in the central cells of the embryo sacs (Figure 3A,B). In *U. humboldtii*, these AGPs occurred in some of the endothelial cells, chalazal cells and in the embryo sac (Figure 3C,D).

**Figure 4:**
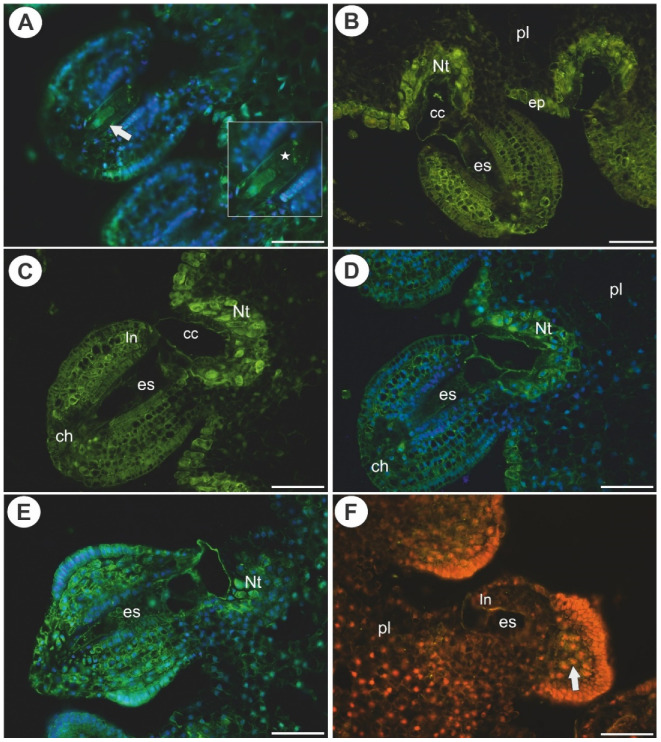
Arabinogalactan proteins (labeled with JIM14 and with LM2) detected in *Utricularia nelumbifolia* and *Utricularia humboldtii* before pollination. (**A**) Arabinogalactan protein (labeled with JIM14, green fluorescence) detected in the ovule of *U. nelumbifolia* that did not contain a mature female gametophyte (arrow). Note the occurrence of AGPs in remaining nucellus epidermis (star, framed part). (**B**–**D**) Arabinogalactan protein (labeled with LM2, green fluorescence) detected in *U. nelumbifolia*; note the strong positive signal in the nutritive tissue (Nt) and epidermis of placenta (ep); embryo sac (es), chalaza (ch), hypertrophied part of central cell (cc), integument (In). (**E**,**F**) Arabinogalactan protein (labeled with LM2, green fluorescence) detected in *U. humboldtii*: embryo sac (es), pl (placenta), nutritive tissue (Nt), positive signal in ovule tissue (arrow); yellowish signal is autofluorescence. All bars 50 µm.

In *Utricularia nelumbifolia*, AGPs (labeled with JIM14) were detected in the integument cells that were located near the micropylar part of the embryo sac as well as in the placenta cells (nutritive tissue), however, only in some cases. These AGPs were also detected in of the embryo sacs in the egg apparatus and central cell (Figure 3E,F). In *U. humboldtii*, these AGPs occurred in the endothelial cells, chalazal cells and placental cells (epidermal cells and nutritive tissue near the embryo sac, Figure 3G,H). In *U. nelumbifolia*, AGPs (labeled with JIM14) were detected in the nucellus in the ovule in which embryo sac development had been arrested (Figure 4A).

In *Utricularia nelumbifolia* and *U. humboldtii*, the AGP epitopes that are recognized by LM2 occurred in the parenchyma cells of the ovules, the cells at the chalaza and in the micropylar part of the embryo sac (Figure 4B–D). These AGPs were also detected in the placental nutritive tissue and epidermis of the placenta of *Utricularia nelumbifolia* (Figure 4E,F).

### 2.2. Observation of Pollen Tube Growth

**Figure 5:**
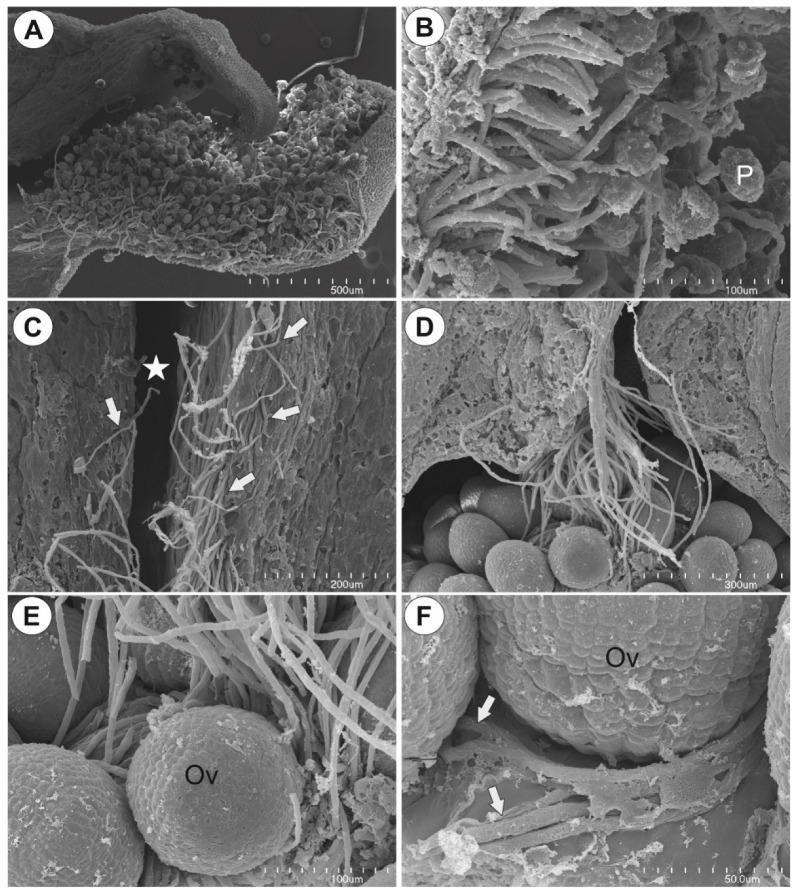
Pollen tube growth in *Utricularia nelumbifolia*. (**A**,**B**) Pollen grains with pollen tubes on the stigma surface; pollen grain (P). (**C**) Pollen tubes (arrows) in the stylar; stylar canal (star). (**D**) Bundle of pollen tubes that entered the ovarian cavity. (**E**) Pollen tubes on the ovules (Ov). (**F**) Pollen tubes (arrows) on the placenta surface: ovule (Ov).

The pollen grains were germinated at the sigma (Figure 5A). Pollen tubes grew on the papillae of the stigma (Figure 5B) and entered the stylar tissue. Next, they entered the stylar canal (Figure 5C). At the bottom of the stylar canal, the pollen tubes formed a bundle that finally entered the ovarian cavity and reached the placenta (Figure 5D). The ovules that were located below ovarian stylar canal were covered by pollen tubes (Figure 5E). Then, pollen tubes grew on the surface of the placenta to reach the embryo sacs of ovules (Figure 5F). Six and nine hours after pollination there were still some nonfertilized ovules. The pollen tubes penetrated the ovules that were located at the top of the placenta first.

### 2.3. Arabinogalactan Proteins in the Placentas and Ovules after Pollination

**Figure 6:**
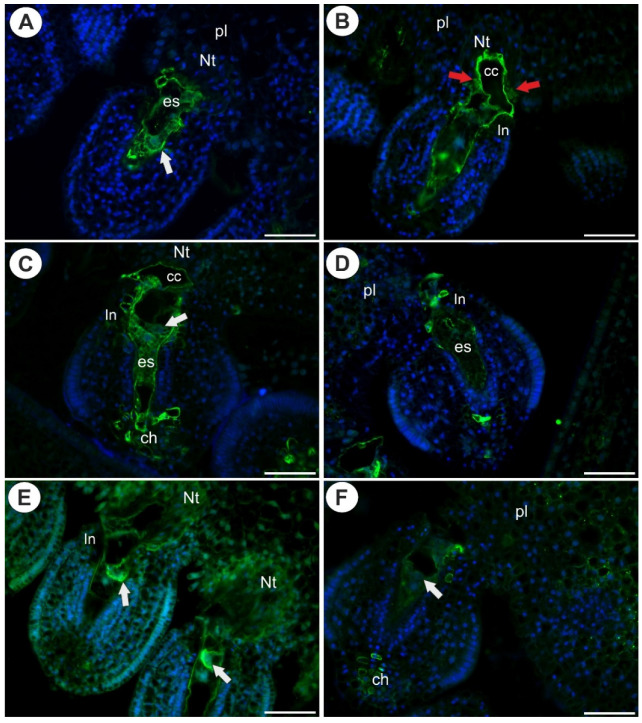
Arabinogalactan proteins detected in *Utricularia nelumbifolia* and *Utricularia humboldtii* after pollination but before fertilization. (**A**,**B**) Arabinogalactan protein (labeled with JIM13, green fluorescence) detected in *U. nelumbifolia*: placenta (pl), placenta nutritive tissue (Nt), embryo sac (es), integument (In), hypertrophied part of the central cell (cc), egg cell (white arrow), positive signal in the placenta epidermal cells (red arrows). (**C**) Arabinogalactan protein (labeled with JIM13, green fluorescence) detected in *U. humboldtii*: nutritive tissue (Nt), hypertrophied part of the central cell (cc), integument (In), embryo sac (es), chalaza (ch). (**D**) Arabinogalactan protein (labeled with JIM8, green fluorescence) detected in *U. humboldtii*: integument (In), placenta (pl), embryo sac (es). (**E**) Arabinogalactan protein (labeled with JIM14) detected in *U. nelumbifolia*: integument (In), nutritive tissue (Nt), egg cell (white arrow). (**F**) Arabinogalactan protein (labeled with JIM14, green fluorescence) detected in *U. humboldtii*: placenta (pl), chalaza (ch), egg cell (white arrow). All bars 50 µm.

**Figure 7:**
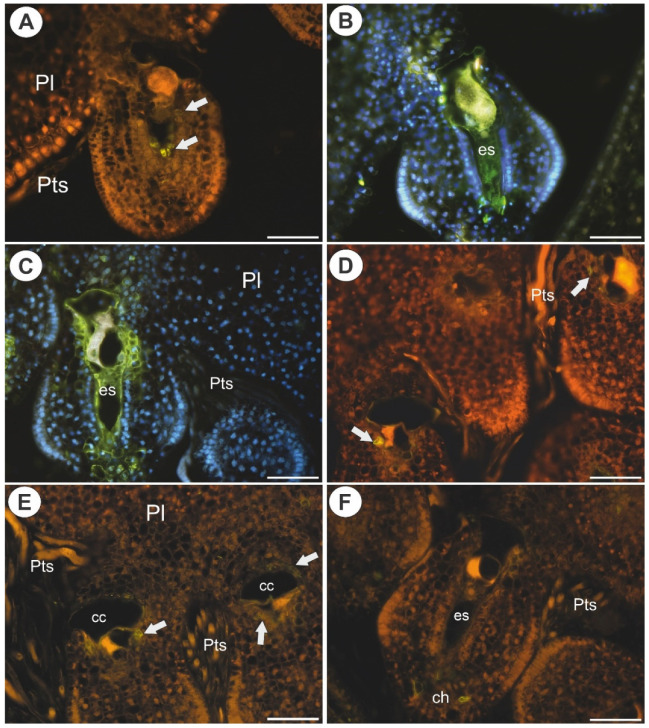
Arabinogalactan proteins detected in *Utricularia nelumbifolia* and *Utricularia humboldtii* after fertilization. (**A**,**B**) Arabinogalactan protein (labeled with JIM13, green fluorescence) detected in *U. nelumbifolia*: placenta (pl), positive signal in the integument cells (arrows), pollen tubes (Pts); yellowish signal is autofluorescence. (**B**,**C**) Arabinogalactan protein (labeled with JIM13, green fluorescence) detected in *U. humboldtii*: embryo sac (es), pollen tubes (Pts), placenta (pl). (**D**) Arabinogalactan protein (labeled with JIM8, green fluorescence) detected in *U. humboldtii*: positive signal in the integument cells (arrows), pollen tubes (Pts); yellowish signal is autofluorescence. (**E**,**F**) Arabinogalactan protein (labeled with JIM14, green fluorescence) detected in *U. humboldtii,* note the positive signal in the integument cells (arrows) and chalaza (ch), pollen tubes (Pts), embryo sac (es), hypertrophied part of the central cell (cc), placenta (pl); yellowish signal is autofluorescence. All bars 50 µm.

**Figure 8:**
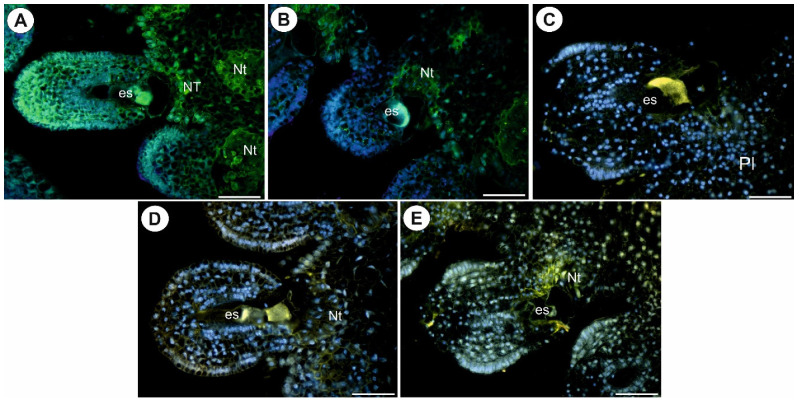
Arabinogalactan proteins (labeled with LM2) detected in *Utricularia nelumbifolia* and *Utricularia humboldtii* after fertilization. Control reactions of the immunolabeling of cell wall components. (**A**,**B**) Arabinogalactan proteins (labeled with LM2) detected in *U. nelumbifolia*; embryo sac (es), nutritive tissue (Nt). (**C**) Arabinogalactan proteins (labeled with LM2) detected in *U. humboldtii*, note no positive signal; embryo sac (es), placenta (Pl); yellowish signal is autofluorescence. (**D**) Ovule of *U. nelumbifolia*; embryo sac (es), nutritive tissue (Nt); yellowish signal is autofluorescence. (**E**) Ovule of *U. humboldtii*; embryo sac (es), nutritive tissue (Nt); yellowish signal is autofluorescence. All bars 50 µm.

As was mentioned earlier, after pollination, there were both fertilized and non-fertilized ovules. In the ovules that had not been penetrated by pollen tubes, the occurrence of AGPs was similar to that in the ovules in the unpollinated flowers (Figure 6A–F). In the fertilized ovules of *Utricularia nelumbifolia* (after pollen tube penetration of synergid), the AGP epitopes that are recognized by JIM13, a positive signal was recoded only in a few cells of the endothelium (Figure 7A). In contrast to *U. nelumbifolia*, in *U. humboldtii*, after pollen tube penetration and fertilization, there was a strong signal in both the female gametophyte and the ovule cells (Figure 7B,C). No AGP epitopes that are recognized by JIM8 were observed in the fertilized ovules of *U. nelumbifolia*. In *U. humboldtii*, these AGPs occurred only in some of the endothelial cells and near the gametophyte (weak signal) (Figure 7D). In *U. humboldtii*, AGPs (labeled with JIM14) were detected in the integument cells that were located near the micropylar part of the embryo sac, the ovular chalazal cells and the placental epidermis (Figure 7E,F). After fertilization, AGPs (labeled with LM2) were detected in the placental nutritive tissue and epidermis of the placenta of *Utricularia nelumbifolia* (Figure 8A,B) in contrast to *U. humboldtii*, where these AGPs were not recorded (Figure 8C).

## 3. Discussion

### 3.1. Occurrence of AGPs before Pollination

Recently, Lora et al. [7] showed that in *Annona cherimola* Mill. and *Arabidopsis thaliana* (L.) Heynh. AGPs occurred in the ovules of both unpollinated and pollinated flowers. Moreover in other angiosperm species, such as *Actinidia deliciosa* (A.Chev.) C.F.Liang and A.R.Ferguson, *Amaranthus hypochondriacus* L. [30], *Olea europaea* L. [39], *Taraxacum officinale* F.H. Wigg. [32,33], *Quercus suber* L. [40], *Fragaria* × *ananassa* Duchesne [41] and *Pilosella officinarum* Vaill. [33], AGPs are produced in the ovule before the pollen tube arrives. However, in other species, the production of AGPs is dependent on pollination and pollen tube growth [7]. We showed that in both examined *Utricularia* species, AGPs were produced in the ovule and placental tissues in the unpollinated flowers, which means that it is not dependent on pollination. When the development of the embryo sac was arrested, AGPs were still produced in the nucellus and integument. Thus, the production of AGPs in sporophytic tissues was not dependent on the occurrence of a mature gametophyte. This result is in contrast to the observation in *Galanthus nivalis* L. that was made by Chudzik et al. [33]. These authors did not find AGPs (labeled by JIM13 and JIM8) in the sterile ovules. Thus, the gametophytic control of the sporophytic tissues may differ from species to species, and in some species, the occurrence of a mature female gametophyte triggers the sporophytic tissues to produce AGPs.

In *U. humboldtii*, an intense accumulation of AGPs (labeled with JIM13) was detected in the chalaza cells. These cells organize the hypostase, which is similar to the hypostase of *Torenia fournieri* [42]. Pereira et al. [43] also found AGPs in the chalazal cells of *Arabidopsis thaliana* ovules.

The AGPs that are labeled by JIM13 and JIM8 are markers for gametophyte cell differentiation [31] and are the most common AGPs that have been reported in female gametophytes by various researchers [28]. Here, we show that in *Utricularia*, AGPs occurred in the egg apparatus and central cell before pollination. Similar results are known for various species, e.g., *Amaranthus hypochondriacus* [30], *Arabidopsis thaliana* [31], *Sida hermaphrodita* (L.) Rusby [44], *Pitcairnia encholirioides* L.B.Sm. [45], *Quercus suber* [46], *Fragaria* x *ananassa* [41] and *Annona cherimola* [7]. There are species-specific differences in AGPs. For example, we noted AGPs that are labeled by LM2 in the central cells, which is in contrast to Mendes et al. [45], who observed these AGPs in most cell types but not in the female gametophyte.

In *Utricularia*, the extra-ovular part of the central cell was rich in AGPs (labeled by JIM13). This result deserves special attention because this part of the female gametophyte is the first to meet the pollen tube.

However, there were differences between *U. nelumbifolia* and *U. humboldtii* for the occurrence of AGPs in the female gametophytes, particularly regarding the AGPs that are labeled by JIM8 and JIM14. Perhaps, these differences are species specific, and they are related to the structure of the nutritive tissues in these species (only in *U. nelumbifolia* did the tissue contain the AGP epitopes that are recognized by LM2). This is especially important because *U. nelumbifolia* and *U. humboldtii* are not closely related, as was shown by studying the phylogeny of *Orchidioides* section [47]. However, more studies that include various species from the same genus are required in order to determine whether production of AGPs is species specific.

### 3.2. AGPs Occurrence after Pollination and Fertilization

Losada and Herrero [6] highlighted the problem that researchers overlook changes in AGPs of the ovules after pollen tube growth. However, Chudzik et al. [34] showed that in *Galanthus nivalis*, pollen tube growth is necessary for the production of AGPs in the ovules. The results of Losada and Herrero [6] for apple ovules are also quite important because these authors showed that AGPs disappeared from some of the ovule tissues following pollen tube passage. These authors also tracked how the pattern of AGP distribution changes during the early stages of the embryo.

Here, we showed that pollination (the occurrence pollen tubes on the placenta surface) did not change the distribution of AGPs in the ovules and female gametophytes in either of the examined species. However, the penetration of the embryo sac by the pollen tube and the process of fertilization changed the pattern of AGPs (labeled by JIM13 and JIM8) distribution in *U. nelumbifolia* dramatically. These AGPs disappeared from the female gametophytes. Additionally, the production of these AGPs was lower in the sporophytic tissues. This is in contrast to *U. humboldtii* in which AGPs occurred abundantly in both the female gametophytes and sporophytic tissues after pollen tube penetration. Thus, changes in the production of AGPs after pollen tube penetration are species specific. According to Losada and Herrero [6], the disappearance of AGPs from the ovule tissues after pollen tube passage proves that there is a pollen-ovule dialogue.

### 3.3. Placental Epidermis and Nutritive Tissue as a Transmission Track for Pollen Tubes

AGPs have been recorded in various unrelated plant species in the cells that form the transmission track for pollen tubes in the tissues of the style, obturator and nucellus e.g., [30,41,48,49,50]. In *Utricularia*, there is special tissue that is called the “nutritive tissue”, “Nährgewebe” or “Drüsengewebe”, that is located in the ovule and/or placenta. This tissue has contact with the embryo sac e.g., [16,51,52,53]. Previous authors have suggested that this nutritive tissue plays a key role in the nutrition of the female gametophyte [16,51,52,53]. In *Utricularia nelumbifolia*, this tissue is well developed, and its cells have nuclei with spindle-like tubular projections (chromatubules) [38]. However, we showed that the AGP epitopes that are recognized by LM2 and JIM14 in the cells of the nutritive tissue and by JIM13 in the epidermal cells are between the nutritive tissue and embryo sac. Therefore, we propose that this tissue may function as an obturator, i.e., it plays a role in pollen tube guidance. In *Utricularia nelumbifolia* and *U. humboldtii*, the pollen tubes grew on the placenta surface in order to reach the ovules. This is in agreement with the observations for other *Utricularia* species [16,54,55].

The occurrence of AGPs in the epidermis of the placenta of *Utricularia* proves that it plays an active role as transmission track for the pollen tubes.

## 4. Materials and Methods

The *Utricularia nelumbifolia* Gardner flowers were obtained in June 2018 and 2019 from the living collections of the Jagiellonian University Botanical Garden in Kraków (Kraków, Poland). The *Utricularia humboldtii* R.H.Schomb. flowers were obtained in April 2020 from the living collections of Department of Plant Ecology, University of Gdańsk (Gdańsk, Poland). The studies were conducted on the flowers during anthesis and in the pollinated flowers that were collected six and nine hours after pollination.

### Sample Preparation

The information on the antibodies that were used in the study to show the distribution of cell wall components is presented in Table 1.

The detailed procedures for observing the histological sections and performing the immunochemical analysis are described in Płachno et al. [33]. The plant material was fixed overnight at 4 °C in 8% (*w/v*) paraformaldehyde (PFA, Sigma-Aldrich, Sigma-Aldrich Sp. z o.o. Poznan, Poland) with 0.25% (*v/v*) glutaraldehyde (GA, Sigma-Aldrich, Sigma-Aldrich Sp. z o.o. Poznan, Poland) in a PIPES buffer (Sigma-Aldrich, Sigma-Aldrich Sp. z o.o. Poznan, Poland). It was then embedded in Steedman’s wax (PEG distearate and 1-hexadecanol; Sigma-Aldrich, Sigma-Aldrich Sp. z o.o. Poznan, Poland) and sectioned into 7 µm sections, which were blocked with 1% BSA in a PBS buffer and incubated with the primary antibodies against arabinogalactans (JIM8, JIM13, JIM14, LM2) overnight at 4 °C. All of the primary antibodies were purchased from Plant Probes, Leeds, UK and the secondary antibody goat anti-rat conjugated with FITC was purchased from Abcam (Abcam plc, registered in England and Wales with Company Number 03,509,322, Discovery Drive, Cambridge Biomedical Campus, Cambridge, CB2 0AX, UK). The chromatin in the nuclei was stained with 7 µg·mL^−1^ DAPI (Sigma-Aldrich, Sigma-Aldrich Sp. z o.o. Poznan, Poland) and the samples were then cover-slipped using a Mowiol medium (Sigma-Aldrich, Sigma-Aldrich Sp. z o.o. Poznan, Poland). They were viewed using a Nikon Eclipse E800 microscope equipped with a B-2A filter, a GFP custom filter, a UV-2A filter and with differential interference contrast (DIC) optics. At least two different replications were performed for each species and developmental stage of the analyzed flowers and about five to ten sections were analyzed from each organ for each antibody that was used. Negative controls were created by omitting the primary antibody step, which caused no fluorescence signal in any of the control frames for any of the stained slides (Figure 8D,E). Decolorized aniline blue (DAB, C.I. 42755, Polyscience Europe GmbH, Hirschberg an der Bergstrasse, Germany) was used to detect the presence of callose in the ovules and placentas. For the SEM, the pistils were fixed in a mixture of 2.5% or 5% glutaraldehyde with 2.5% formaldehyde in a 0.05 M cacodylate buffer (Sigma-Aldrich, Sigma-Aldrich Sp. z o.o. Poznan, Poland; pH 7.2) overnight, washed three times in a 0.1 M sodium cacodylate buffer and later dehydrated and critical point dried using CO_2_. They were then sputter-coated with gold and examined at an accelerating voltage of 20 kV using a Hitachi S-4700 scanning electron microscope (Hitachi, Tokyo, Japan), which is housed in the Institute of Geological Sciences, Jagiellonian University in Kraków, Poland.

## 5. Conclusions

1. In both of the examined species, arabinogalactan proteins (AGPs) were observed in the placenta, ovule (integument, chalaza) and female gametophyte of both pollinated and unpollinated flowers, thus the production of AGPs was not dependent on pollination; however, the production of some AGPs was lower after fertilization.
2. The production of some AGPs in the ovular tissues (nucellus, integument) was not dependent on the presence of a mature embryo sac. Thus, gametophytic control of the sporophytic tissues may differ from species to species.
3. The occurrence of AGPs in the placenta epidermis and nutritive tissue of *Utricularia* supports the hypothesis that they play an active role as a transmission track for pollen tubes.
4. In recent years, *Utricularia* has been proposed as a model to study plant genome evolution e.g., [58,59,60,61], carnivory and plant development e.g., [62,63,64,65,66,67,68,69]. Here, we showed that *Utricularia* can also be a useful and attractive model to study the male–female dialogue in plants.
5. We plan to study cell wall components in ovules and placentas of other *Utricularia* species that have differently developed nutrient tissues and form special syncytia.

## Figures and Tables

**Table 1:** Monoclonal antibodies that were used in the current study, the epitopes that they recognize and references.

Antibody	Epitope	References
JIM8	Arabinogalactan	[56]
JIM13	Arabinogalactan/arabinogalactan protein	[57,58]
JIM14	Arabinogalactan/arabinogalactan protein	[57,58]
LM2	Arabinogalactan/arabinogalactan protein	[56]

## Data Availability

Not applicable.
